# Deciphering the effect of novel bacterial exopolysaccharide-based nanoparticle cream against *Propionibacterium acnes*

**DOI:** 10.1007/s13205-015-0359-5

**Published:** 2016-01-23

**Authors:** Abraham P. Karlapudi, Vidya P. Kodali, Krishna P. Kota, Sabiha S. Shaik, N. S. Sampath Kumar, Vijaya R. Dirisala

**Affiliations:** 1 Department of Biotechnology, Vignan’s University, Guntur, 522213 AP India; 2 Department of Biotechnology, Vikrama Simhapuri University, Nellore, 524003 India

**Keywords:** *Propionibacterium acnes*, *Acne vulgaris*, EPS nanoparticles, Antibacterial activity

## Abstract

*Acne vulgaris* (acne) is a chronic inflammatory disease prevalent among adolescents and adults, with significant psychological effects. The aetiology of acne is multifactorial. Several pathophysiological associations have been identified in which *Propionibacterium acnes* plays a major role. This bacteria primarily affects areas containing oil glands including the face, back and trunk, where it causes the formation of seborrhoea and inflammatory lesions. The treatment methods currently in place have side effects. A novel alternative method with no side effects is hence required. In this study, we report the synthesis of an exopolysaccharide (EPS)-producing bacterial-based nanoparticle as a stable biocompatible material for drug delivery. We then evaluated the effectiveness of EPS-based nanoparticle cream against *P. acnes*. Our results demonstrate that EPS nanoparticles have great potential as a safe and effective topical treatment for *acne vulgaris* and other associated infections.

**Electronic supplementary material:**

The online version of this article (doi:10.1007/s13205-015-0359-5) contains supplementary material, which is available to authorized users.

## Introduction

*Acne vulgaris* (acne) is a chronic inflammatory disease of the sebaceous gland characterized by follicular hyperkeratinization and excessive colonization by *Propionibacterium acnes* (*P*. *acnes*) (Beylot et al. 2014). Acne affects the majority of adolescents, who develop inflammatory lesions such as papules, pustules and nodules, as well as other non-inflammatory lesions (Webster 2014). *P. acnes* activates immune reactions by releasing chemo-active agents that attract neutrophils, monocytes, lymphocytes, and stimulate the production of pro-inflammatory cytokines (Kistowska et al. 2015). Despite the availability of a number of antibiotics, antibiotic resistant strains of acne are a cause of major global concern (Lomholt and Kilian 2014). In consequence, there have been increased attempts to find a naturally occurring anti-acne agent (Tin and Wiwanitkit 2014). Over the past few decades, exopolysaccharides have gained attention for their bioactive properties such as stability, biodegradability and biocompatibility. In general, exopolysaccharides (EPS) are usually anionic heteropolysaccharides due to the presence of various constituents such as xanthan, alginate and glycans, etc. EPS produced by microorganisms vary in their composition and properties such as anti-bacterial, anti-cancer, anti-tumoral, anti-ulcer, anti-immune stimulation along with cholesterol-lowering ability (Kumar et al. 2007; Raveendran et al. 2013; Raposo et al. 2014). The mechanism of EPS owing to the antimicrobial activity is attributed to its low degree of polymerization and degree of deacetylation (Poli et al. 2011). EPS penetrates into the cell membrane of the bacteria which in turn interrupts the replication process of bacteria there by suppressing microbial growth (Nwodo et al. 2012). In this study, we synthesized an exopolysaccharide (EPS)-producing bacterial-based nanoparticle serving as a stable biocompatible material for drug delivery. We then evaluated the effectiveness of the EPS-based nanoparticle cream’s antimicrobial activity against *P. acnes.*

## Materials and methods

### Isolation studies of the *P. acnes* strain

Isolation of *P. acnes* from the human skin was performed using nutrient broth (HiMedia, India) and Trypticase Soy Agar (HiMedia, India) with 5 % sheep blood on petri plates. Different facial samples of 1 cm^2^ were taken from 22 healthy human volunteers. These were cleansed with a sterile swab and placed in 5 mL of phosphate buffer saline (Life Technologies, USA). Dilutions were made, and 100 µl of each sample was plated then incubated anaerobically at 37 °C for 48 h. After 48 h of growth, biochemical tests were performed to determine the presence of *P. acnes.* The reference strain used in our study was *P. acnes*, microbial type culture collection (MTCC) no: 1951. Consent was obtained from patients who took part in this study and all ethical procedures were strictly followed.

### EPS extraction

EPS was extracted from a biofilm-producing *Acinetobacter Sea*-*9* bacterium (Kodali and Sen 2008; Sujana et al. 2013) by centrifuging the overnight bacterium culture at 10,000 rpm for 20 min at 4 °C to remove the bacterial cells. The obtained supernatant was collected in a fresh vial and precipitated with two volumes of ice-cold absolute ethanol by incubating the mixture at 4 °C overnight. The mixture was then centrifuged at 10,000 rpm for 20 min at 4 °C the following day. The supernatant was discarded, and the pellet containing EPS was dried at room temperature. It was then dissolved in pure distilled water, and purified for 48 h by dialysis, using dialysis tubing with a 10,000 molecular weight cut-off. Finally, the purified EPS was freeze-dried, and characterized by Fourier transform infrared spectroscopy [FTIR (Mancuso Nichols et al. 2004)]. The total carbohydrate concentration was determined using phenol–sulphuric acid method (DuBois et al. 1956).

### Synthesis of EPS nanoparticles

As reported previously by others, chitosan molecules undergoing poly-electrolyte complexation with polysaccharides can spontaneously give rise to nanoparticles under strong magnetic stirring (Fotticchia et al. 2014). Various concentrations of EPS solutions were hence mixed with chitosan solution under strong magnetic stirring to form nanoparticles. EPS nanoparticles were prepared by complex formation between EPS and chitosan under strong magnetic stirring. Chitosan was dissolved in 1 % (w/v) acetic acid to obtain a final strength of 10 mg/mL. EPS concentrations were prepared in distilled water, as well as different solutions with 1–5 mg/mL concentrations at different pHs. Suspensions of nanoparticles formed spontaneously when chitosan solution was added to EPS solution under strong magnetic stirring at room temperature with varying pHs. Nanoparticle suspensions were filtered and mixed with cream formulation to check the antimicrobial properties (Sivakumar et al. 2014; Sathiyanarayanan et al. 2014).

### EPS nanocream formulation

For the oil phase, 1.85 g of beeswax was added to 5 mL of liquid paraffin, followed by a few drops of glycerine. For the aqueous phase, 0.15 g of borax was added to 3 mL DH_2_O. The cream was obtained by stirring the two phases continuously until they reached 70 °C. The emulsion of EPS nanoparticles and cream was prepared by adding 1 mL of the EPS solution at different concentrations (1–5 mg/mL). This was then left overnight to test for stability. The antimicrobial effect of the selected strain was tested using the agar well diffusion method (Bonev et al. 2008).

## Results

### Isolation studies of the *P. acnes* strain

**Fig. 1:**
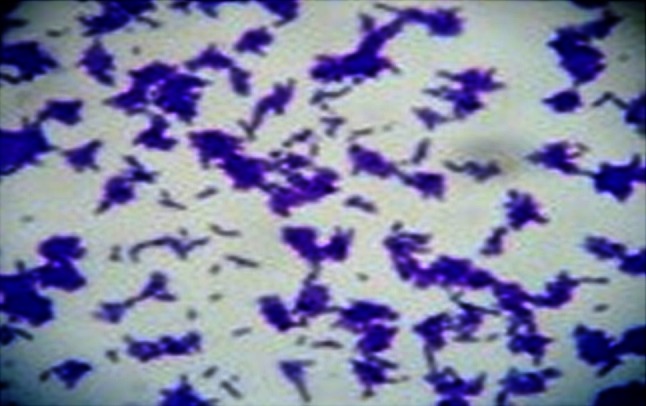
Gram staining of S19 bacteria. Staining confirmed that S19 is gram positive and rod shaped

Twenty-two pure colonies were isolated, of which four samples numbered S4, S7, S14 and S19 were screened biochemically to identify the *P. acnes* strain. S19 showed similar biochemical characteristics to reference strain *P. acnes* MTCC 1951 (Fig. 1; Supplementary Table 1).

### EPS analysis

FTIR analysis of extract from *Acinetobacter Sea*-*9* bacterium showed the presence of –OH band at 3346.62 cm^−1^ and COOH groups at 1637.86 cm^−1^ confirming that the sample was EPS. On chemical analysis using the phenol–sulphuric acid method, it was found that the total carbohydrate content in EPS extract was 78 μg/mL.

### Synthesis of EPS nanoparticles

The concentration and pH of chitosan and EPS solutions plays a crucial role in the formation of nanoparticles (Raveendran et al. 2013). Combinations of different pH were hence evaluated to determine the optimal level for the formation of stable nanoparticles. When the pH of chitosan solution was in the range of 3, and EPS solution was in the range of 4.5, stable nanoparticles were formed. Gradual increases or decreases in the pH values of EPS and chitosan solutions outside the above-stated optimal ranges resulted in precipitate formation. Combinations of various concentrations of chitosan and EPS solutions were also analysed for nanoparticle formation. When the concentration of chitosan was 6 mg/mL, and the EPS concentration varied between 1 and 7 mg/mL, an increase in the size of the particles was observed. Large aggregates were formed when the ratio was 6:6. Upon increasing the ratio to 6:7, macro-globules formed that were discernible to the naked eye.

### Antibacterial activity of the EPS nanoparticles

**Fig. 2:**
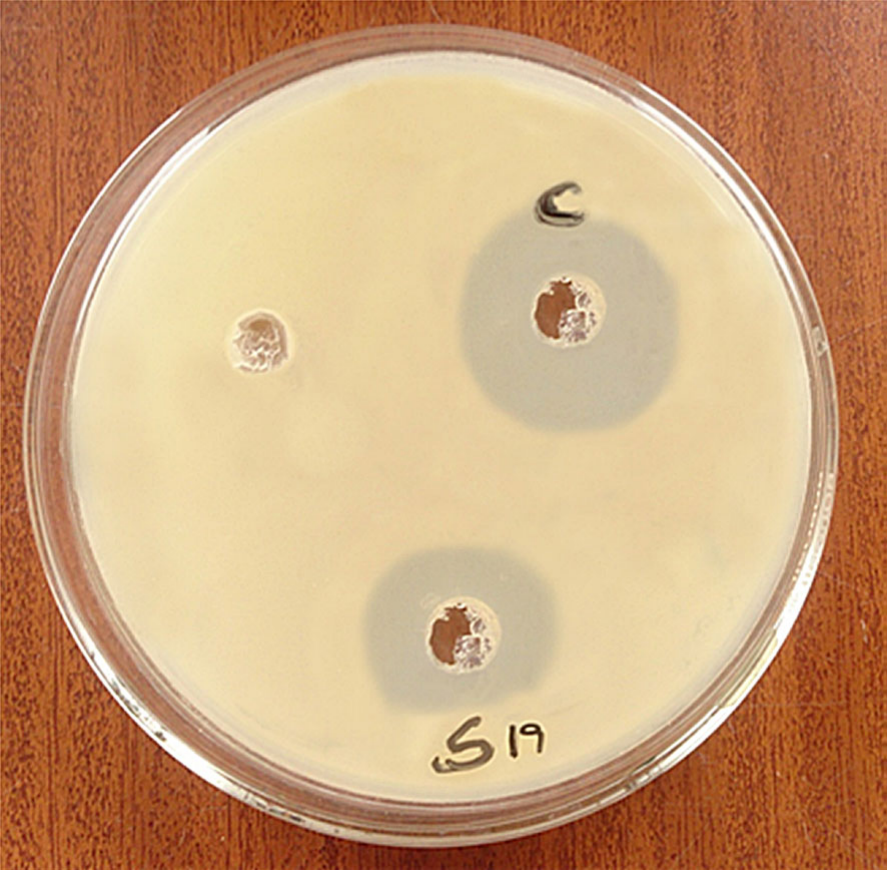
Antibacterial activity of EPS nanoparticles in cream against sample 19 (S19) at 39 μg/mL concentration [Control (C)—tetracycline]; (no name—chitosan + cream)

The antibacterial activity of EPS nanoparticles against S19 strain was performed by checking varying concentrations. The EPS nanoparticles were effective against *P. acnes* at a concentration of 39 μg/mL, and the zone of inhibition was measured as 18 mm (Fig. 2). Cream with and without chitosan did not show any activity against the organism. After 2 weeks, the EPS nanoparticles were found to be quite stable in a cream emulsion. It was observed that the zone diameter measured for the activity of EPS nanoparticles emulsified in cream was more or less same as that of solitary EPS nanoparticles (18 and 18.5 mm against S19, respectively). This proves that EPS nanoparticles are stable in emulsions. The antibiotic tetracycline was also effective against the organism, with a zone diameter of 24 mm (Fig. 2). However, literature suggests that the organism has shown resistance to such antibiotics (Simonart et al. 2008; Andriessen and Lynde 2014). A novel treatment should hence involve a substance which is less toxic but more effective.

## Discussion

Acne is a chronic inflammatory disease which is prevalent among adolescents and adults with significant psychological effects (Durai and Nair 2015). Acne-related psychological suffering could be consequently linked to the occurrence of psychiatric disorders such as anxiety, depression, and suicidal ideation (Pace 2014). Standard oral and topical therapies which are being administered are accompanied with side effects such as skin irritation, gastrointestinal upset, and the development of drug-resistant bacteria (Lauren 2014). Research suggests that many patients are vying towards safe alternative therapies for treatment of acne. Considering that exopolysaccharides are accompanied with no side effects (van Bueren et al. 2015), we synthesized and evaluated the effectiveness of EPS-based nanoparticle cream against *P. acnes*. These findings have opened new avenues to explore the significant role of EPS nanoparticles as a safe and effective topical treatment for *acne vulgaris* and other associated infections.

## Electronic supplementary material

Below is the link to the electronic supplementary material.

Supplementary material 1 (DOCX 14 kb)
