# Supplementary material for: Deciphering the effect of novel bacterial exopolysaccharide-based nanoparticle cream against Propionibacterium acnes
Source: 3 Biotech. 2016 Jan 23;6(1):35. doi: 10.1007/s13205-015-0359-5 (PMC4724354; doi:10.1007/s13205-015-0359-5)
Supplement: Supplementary file 1 — Supplementary material 1 (DOCX 14 kb) [file 13205_2015_359_MOESM1_ESM.docx]

| **S. no** | **Biochemical tests** | **Sample 19** | ***P.acnes***  ***MTCC 1951*** |
| --- | --- | --- | --- |
| **1.** | **Urease** | **Positive** | **Positive** |
| **2.** | **Carbohydrate metabolism**   1. **Lactose** 2. **Sucrose** 3. **Glucose** 4. **Xylose** 5. **Arabinose** | **Positive**  **Positive**  **Positive**  **Positive**  **Negative** | **Positive**  **Positive**  **Positive**  **Negative**  **Negative** |
| **3.** | **Citrate utilization** | **Positive** | **Positive** |
| **4.** | **H_2_S production** | **Negative** | **Negative** |
| **5.** | **Starch hydrolysis** | **Positive** | **Positive** |
| **6.** | **Indole production** | **Negative** | **Negative** |
| **7.** | **MR** | **Negative** | **Negative** |
| **8.** | **VP** | **Negative** | **Negative** |
| **9.** | **Catalase** | **Positive** | **Positive** |
| **10.** | **Lipase** | **Positive** | **Positive** |
| **11.** | **Gelatin Hydrolysis** | **Positive** | **Positive** |
| **12.** | **Nitrate Reduction** | **Positive** | **Positive** |
